# "Is Cybermedicine Killing You?" - A Response From the Authors of the Cochrane Review: Author's Reply (1)

**DOI:** 10.2196/jmir.7.4.e41

**Published:** 2005-07-28

**Authors:** Roy Rada

## Author's Response

Murray et al's letter to the editor says that my paper [1] contains three factual inaccuracies. The first is that Nazareth was not responsible for coding the data and is not credited with this in the review. In fact, the list of contributions in the review did not explicitly use the term "coding," but it credits Tai with "designed analytical strategy, extracted and synthesised data...," and Nazareth with "designed review and analytical strategy, interpreted data..." from which I concluded that both Nazareth and Tai were jointly responsible for coding. I appreciate the clarification of Murray and colleagues. Secondly, my statement that the "UCL...news bulletin...remained there" was not meant to imply that it remained there devoid of a retraction statement. In fact, I did write that "the original press releases [are] now marked with 'retraction.'" Regarding the third issue, I acknowledge that this has been an oversight on my part. I apologize for interpreting incorrectly for item 1, communicating ambiguously for item 2, and being factually wrong for item 3.
